# Transcriptomic Analysis of Immune Tolerance Induction in NOD Mice Following Oral Vaccination with GAD65-*Lactococcus lactis*

**DOI:** 10.3390/vaccines13090927

**Published:** 2025-08-30

**Authors:** Mengxin Xie, Chunli Ma, Xinyi Wang, Tengjiao Li, Shihan Zhang, Jiandong Shi, Jing Sun, Yunzhang Hu

**Affiliations:** 1 Institute of Medical Biology, Chinese Academy of Medical Sciences and Peking Union Medical College, Kunming 650118, China; oho_teemo@yeah.net (M.X.); wxyvicky@yeah.net (X.W.); litengjiao@stu.ynu.edu.cn (T.L.); zsh15226927851@163.com (S.Z.); shijiandong@imbcams.com.cn (J.S.); 2 Kunming Medical University, Kunming 650031, China; 20191167@kmmu.edu.cn; 3 Department of Medical Laboratory, The Third People’s Hospital of Honghe, Gejiu 661000, China; 4 School of Food and Pharmacy, Yuxi Vocational and Technical College, Yuxi 653100, China; 5 School of Life Sciences, Yunnan University, Kunming 650091, China

**Keywords:** type 1 diabetes, oral vaccine, immune response, transcriptomic response

## Abstract

Background: Type 1 diabetes (T1D) is an autoimmune disorder characterized by destruction of insulin-producing β-cells. While conventional insulin therapy manages hyperglycemia, it fails to halt autoimmunity. Oral immunotherapy targeting autoantigens like GAD65 offers potential for antigen-specific tolerance; however, its efficacy is limited by gastrointestinal degradation and poor mucosal uptake. *Lactococcus lactis* (*L. lactis*), a food-grade delivery vector, enables sustained antigen release and intestinal tract immune modulation, yet the differential transcriptomic mechanisms underlying mucosal versus systemic immune responses remain uncharacterized. Methods: Non-obese diabetic (NOD) mice were randomized into control and GAD65 groups, receiving oral PBS or the GAD65 recombinant *L. lactis* vaccine, respectively. Fasting blood glucose was monitored weekly. GAD65-specific IgA and IgG, along with immune tolerance-related factors, were quantified using ELISA. Lymphocyte subsets were analyzed by flow cytometry, alongside RNA sequencing and transcriptional profiling. Results: The study demonstrated that the orally administered GAD65-*L. lactis* vaccine could significantly induce GAD65-specific IgA antibody and TGF-β cytokine and alleviate hyperglycemia and diabetes symptoms in NOD mice. Our study facilitated the induction of GAD65-specific regulatory T cells within both intestinal lamina propria lymphocytes (LPLs) and splenic lymphocytes. Notably, antigen-specific tolerance was mainly observed in intestinal LPLs. Crucially, the immune responses elicited by the vaccine demonstrated significant disparities between intestinal LPLs and splenic lymphocytes, with intestinal LPLs exhibiting unique local immune tolerance transcriptomic profiles. Conclusions: Our findings have enhanced the comprehension of the mechanisms by which oral vaccines influence the interplay between mucosal and systemic immune responses, thereby establishing a foundational framework for the design of oral vaccines. This understanding is instrumental in advancing antigen-specific immune tolerance strategies for autoimmune diseases such as Type 1 Diabetes (T1D).

## 1. Introduction

Type 1 diabetes (T1D) is a chronic autoimmune disorder characterized by the destruction of insulin-producing pancreatic β-cells due to an attack by autoreactive T cells. Advances in sustained-release insulin pumps, genetically engineered long-acting insulin, and the anti-CD3 monoclonal antibody teplizumab have improved hyperglycemia management in T1D patients. However, the high cost and potential side effects of these treatments pose significant challenges.

Autoantibodies such as insulin (IA), glutamic acid decarboxylase (GAD65), insulinoma antigen (IA-2), and islet zinc ion transporter (ZnT8) have been detected in individuals with T1D months or even years before clinical onset [1]. Therefore, developing novel immunotherapies that elicit potent and selective antigen-specific tolerance is a promising approach for treating autoimmune diseases [2]. Previous studies have demonstrated that vaccines containing the GAD65 protein or DNA plasmids can induce protective immune tolerance, preventing and ameliorating T1D in NOD mice [3,4]. The GAD65-alum vaccine, developed by Diamyd Medical in Stockholm, Sweden, is presently in phase III clinical trials. However, it was subsequently found that the vaccine could only be used to treat T1D in pediatric patients with stage 1 or 2 T1D who carried the HLA DR3-DQ2 genotype [5].

Oral antigen administration can induce specific immunological hyporesponsiveness, known as oral tolerance [6]. The U.S. Food and Drug Administration (FDA)-approved peanut allergy drug Palforzia exemplifies a successful tolerance therapy using this approach [7]. The mechanism involves regulatory T cells (Tregs) secreting IL-4, IL-10, and transforming growth factor-β (TGF-β), which promote systemic immune tolerance through cytokine-mediated bystander suppression [8]. Therefore, oral vaccines designed to induce tolerance may offer an effective strategy for T1D prevention, particularly in children and the elderly.

However, the intestinal tract significantly reduces the efficacy of orally administered soluble proteins in inducing immune tolerance. To overcome this, the food-grade bacterium *L. lactis* has been engineered for mucosal antigen delivery. Its GRAS (Generally Recognized as Safe) status [9,10], genetic tractability, and ability to enhance antigen presentation in GALT make it an ideal vector [11,12]. In fact, a large number of studies have utilized *L. lactis* as a carrier to deliver antigens for the treatment of diseases and have achieved satisfactory results [13,14]. Robert et al. demonstrated that oral *L. lactis*-expressing GAD65 suppresses diabetes in NOD mice by inducing Tregs [15]. In addition, Mathieu et al. demonstrated that oral delivery of AG019, a food-grade *L. lactis* genetically engineered to express human proinsulin and human IL-10, was well tolerated and safe as monotherapy and in combination with teplizumab [16].

In this study, our findings demonstrate that oral administration of the GAD65-*L. lactis* vaccine effectively induces antigen-specific regulatory T cells and tolerance, enhances TGF-β secretion, and mitigates T1D development in NOD mice. Notably, the immune response elicited by this vaccine differed markedly between intestinal LPLs and splenic lymphocytes, a distinction that has not been comprehensively characterized in prior studies.

In the meantime, the transcriptomic mechanisms that critically drive the compartmentalized immune response between intestinal LPLs and splenic lymphocytes remain to be elucidated. To investigate these compartment-specific differences, we conducted a comparative transcriptomic analysis of intestinal LPLs and splenic lymphocytes using RNA sequencing. Analyses of Gene Ontology (GO), Kyoto Encyclopedia of Genes and Genomes (KEGG), and Gene Set Enrichment Analysis (GSEA) pathways revealed significant divergences in cell adhesion, amino acid metabolism, immune responses, and inhibitory inflammatory cytokines. Additionally, we systematically compared genes related to T and B cell functions—including signaling pathways, cytokines, surface markers, and transcription factors—between these compartments. Our findings revealed a transcriptomic profile predominantly characterized by mucosal immune tolerance, with secondary effects on systemic immune regulation. These results offer novel insights into the mechanisms underpinning the efficacy of oral vaccines for T1D. This understanding is crucial for advancing antigen-specific immune tolerance strategies for autoimmune diseases, including T1D.

## 2. Materials and Methods

### 2.1. Ethics Statement

This study and all related procedures were approved by the Ethics Committee of the Institute of Medical Biology, Chinese Academy of Medical Sciences (Approval ID: DWSP202004 028). All animal handling protocols adhered to ethical guidelines for the care and use of animals in scientific research. The animals were obtained from Beijing HFK BIOSCIENCE Co., Ltd. (Beijing, China).

### 2.2. Animals, Experimental Design, and Sampling

Six-week-old female NOD mice were randomly assigned to two groups (eight per group). The GAD65 group received an oral immunization of *L. lactis* recombinant GAD65 vaccine (10^9^ CFU/mouse; 100 µL, 10^10^ CFU/mL), while the control group received 100 µL PBS via the same route. All mice were orally immunized for 7 consecutive days, followed by another 7 consecutive days at an interval of 2 weeks, and the food was stored away 6 h before immunization. GAD65: recombinant GAD65 *L. lactis* vaccine; Control: PBS. Twelve hours after the last immunization, 3 mice were sacrificed for the colonization experiment and the remaining 5 mice were observed until week 14.

**Figure 1:**
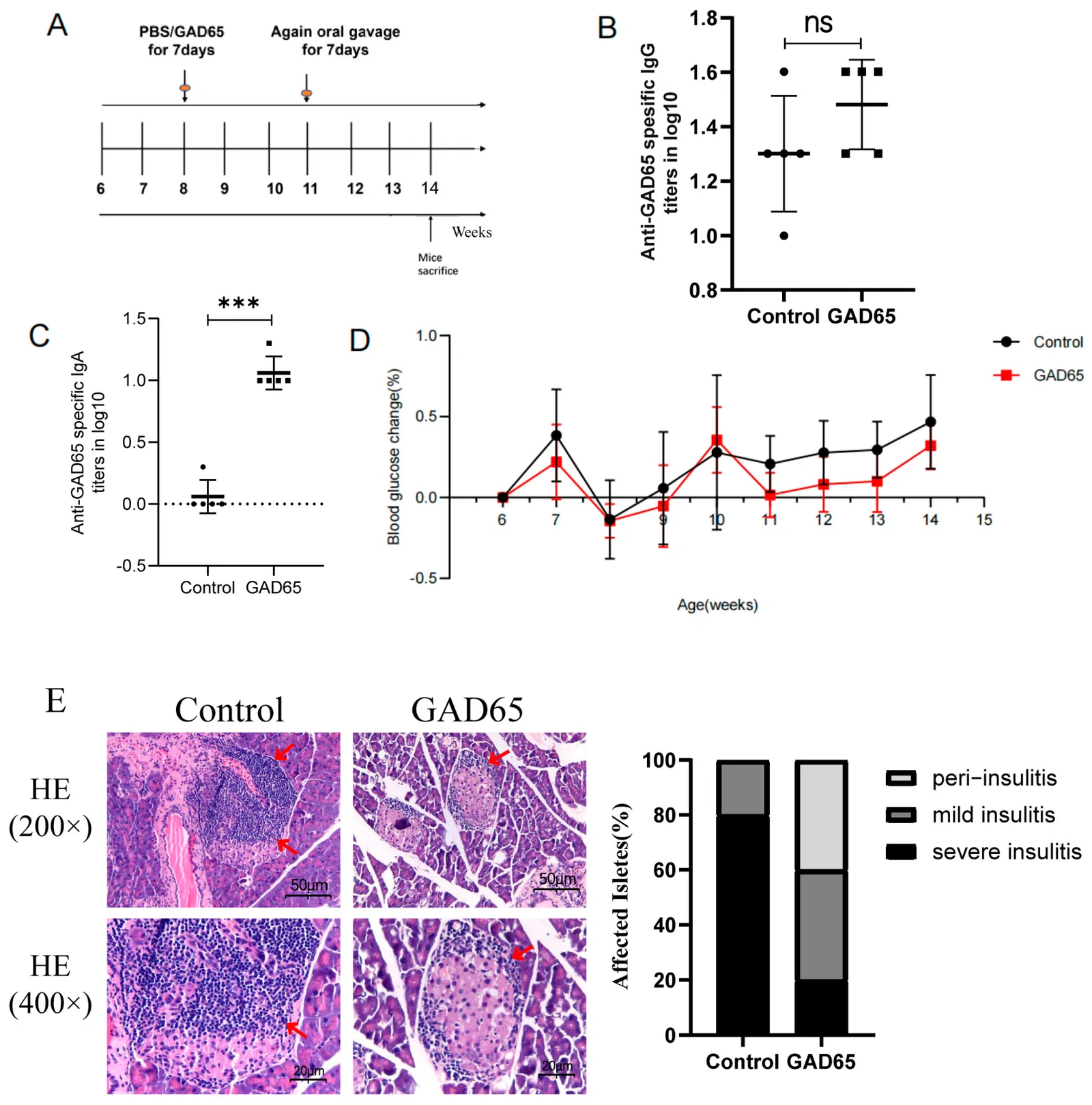
Oral immunization schedule and detection of physiological indicators related to T1D in NOD mice. (**A**) Oral immunization schedule and time points of sacrificed mice for sampling (*n* = 8). (**B**) GAD65-specific IgG titers in mice serum (*n* = 5, ns *p* > 0.05). (**C**) GAD65-specific sIgA titers in mice serum (*n* = 5, *** *p* < 0.005). (**D**) Glucose changes in NOD mice at 6–14 weeks of age (*n* = 5). (**E**) Histopathological sections and severity of islet inflammation in NOD mice two weeks after the last oral immunization. The area indicated by the arrow represents the presence of inflammatory infiltration (*n* = 5).

In a previous study, we constructed a full-length, human codon-optimized GAD65 gene (GenBank: M81882.1) on an *L. lactis* vector, producing both secreted and cytoplasmic GAD65 protein expression (Supplementary Figure S1A,B). To ascertain the survivability of *L. lactis* within the intestinal tract, intestinal contents from NOD mice were collected and serially diluted with PBS from 10^−1^ to 10^−10^ after 12 h of the last immunization. The resulting dilutions were then cultured on solid GM medium (M17 agar supplemented with 0.5% glucose) containing chloramphenicol (20 µg/mL) at 30 °C under anaerobic conditions. (Supplementary Figure S1C). Six-hour fasting blood glucose levels of NOD mice were monitored, and splenic lymphocytes and intestinal LPLs were collected (Figure 1A). RNA sequencing samples were prepared, and serum and fecal samples were stored at −80 °C until analysis. The schedule for immunization and sample collection is as described earlier.

### 2.3. ELISA

#### 2.3.1. GAD65-Specific IgA and IgG Detection

Flat-bottom 96-well plates (Costar, Corning, ME, USA) were coated overnight at 4 °C with purified recombinant GAD65 protein (Proteintech, Rosemont, IL, USA) at a concentration of 0.01 mg/mL in coating buffer (0.012 M Na_2_CO_3_ and 0.038 M NaHCO_3_, pH 9.6). Plates were washed five times with PBST and then blocked with 1% BSA in PBST at 37 °C for 1 h. Mouse fecal diluent samples were serially diluted twofold in blocking solution (starting at 1:2) and added to wells (100 µL per well). After incubation at 37 °C for 1 h, plates were washed five times with PBST, followed by incubation with HRP-labeled goat anti-mouse IgA antibody (Proteintech, Rosemont, IL, USA) diluted 1:1000 at 37 °C for 1 h.

Following the final wash, fresh TMB substrate from the ELISA Kit Anti-Mouse ABTS System (KPL, Milford, MA, USA) was added (100 µL per well) and incubated for 5 min. The reaction was stopped with 25 µL of 2M H_2_SO_4_, and the optical density (OD) was measured at 405 nm using a multifunction microplate reader (FlexStation 3, San Jose, CA, USA).

The method for detecting GAD65-specific IgG is the same as described above, but the HRP-labeled goat anti-mouse IgA antibody used therein needs to be replaced with HRP-labeled goat anti-mouse IgG antibody (KPL, Milford, MA, USA).

#### 2.3.2. Serum TGF-β Detection

TGF-β levels were measured using the Human/Mouse TGF-β1 Uncoated ELISA Kit (Thermo Scientific, Vienna, Austria) according to the manufacturer’s instructions. Plates were prepared and sealed, and standards and acid-activated serum samples were processed accordingly.

Activated NOD mouse serum samples (100 µL per well) were added and incubated at room temperature for 2 h. Wells were aspirated and washed three times with >250 µL/well wash buffer. The detection antibody was prepared by diluting 250× detection antibody with 1× ELISA spot diluent and adding 100 µL per well, followed by incubation at room temperature for 1 h.

After washing, 1× Avidin-HRP (100 µL per well) was added and incubated at room temperature for 30 min. Following the final wash, 1× TMB substrate (100 µL per well) was added and incubated at room temperature for 15 min before stopping the reaction with the termination solution. Absorbance (OD) was measured at 450 nm using a multifunction microplate reader (FlexStation 3, San Jose, CA, USA).

### 2.4. Flow Cytometry Assay

Mice splenocytes were harvested after fourteen days of the last immunization. Splenocytes were recovered as a single-cell suspension in RPMI 1640 medium (Hyclone, Marlborough, MA, USA). The erythrocytes were removed from splenic suspensions using 1X RBC lysis buffer (eBioscience, Carlsbad, CA, USA) for 5 min at room temperature. Then, the RPMI 1640 complete culture medium supplemented with 10% FBS (BI, kibbutz, Israel) was added. Cell viability and concentration were assessed by mixing 10 μL trypan blue with 10 μL cell suspension and analyzing via an automated cell counter (Bio-Rad, Hercules, CA, USA), with the final concentration adjusted to 5 × 10^6^ cells/mL. The purity and viability of the cells were maintained at a minimum threshold of 90%.

Intestinal LPLs were isolated using a modified protocol as previously outlined by Lee JS et al. [17]. Initially, the small intestine was incised and immersed in ice-cold PBS for washing. The intestine was opened longitudinally, sectioned into 2 cm^2^ pieces, and transferred to an EDTA-containing stripping buffer (Hank’s balanced salt solution (HBSS) supplemented with 5% FBS, 2 mM EDTA, 10 mM HEPES, and 1 mM/L DTT). The tissue was incubated at 37 °C for 30 min on a shaking platform. Following incubation, the supernatant was filtered through a 70 µ nylon cell strainer to collect the intestinal fragments. This procedure was repeated twice. Subsequently, the intestinal segments were immersed in a digestion buffer (PBS supplemented with 5% FBS, collagenase type IV (1.5 g/L, Sigma, Saint Louis, MO, USA), and DNase I (100 Ku/L, Sigma, Saint Louis, MO, USA). The mixture was incubated for 45 min at 37 °C with continuous agitation. Following this, the suspension containing lamina propria lymphocytes (LPLs) was retrieved. LPLs were then isolated by density gradient centrifugation using a Percoll buffer (Sigma, Saint Louis, MO, USA). At last, the RPMI 1640 complete culture medium supplemented with 10% FBS was added. Using the method described earlier to determine the cell viability and concentration, ensure that the purity and viability of the cells remain at the minimum threshold of 70%. Subsequent antigen stimulation was performed under identical culture conditions.

For antigen stimulation, GAD65 peptides (the peptides were synthesized by Shanghai Anlian Peptide Co., Ltd., Shanghai, China. with a purity of over 95%) (I. TYEIAPVFVLLEYVT; II. EYVTLKKMREIIGWPGGSGD; III. KKGAAALGIGTDSVI; IV. ALGIGTDSVILIKCDERGK; V. TLEDNEERMSRLSK) were dissolved in RPMI 1640 medium supplemented with 10% FBS to achieve a final concentration of 10 μg/mL. Aliquots of 100 μL peptide solutions were distributed into designated wells of a U-bottom 96-well plate. Subsequently, 100 μL of an adjusted cell suspension was added to each well, resulting in a final volume of 200 μL per well. The cultures were incubated at 37 °C with 5% CO_2_ under gentle agitation for a duration of 48 h.

The following fluorescently labeled anti-mouse monoclonal antibodies were used: anti-mouse CD4-FITC (GK1.5), anti-mouse CD25-PE (PC61.5), anti-mouse/Rat-Foxp3 PE (FJK-16s), anti-mouse CD11c-PE (N41 8), anti-mouse CD80-APC (16-10A1), anti-mouse IL-10-FITC (JESS-16E3), and anti-mouse CD8a-PE (53-6.7). Carboxyfluorescein succinimidyl ester (CFSE) was also used. Following staining and washing, samples were resuspended in Permeabilization Buffer and immediately analyzed using the CytoFLEX-S flow cytometer (Beckman Coulter, Brea, CA, USA). The flow data were processed using CytExpert software 2.3.1.22. All reagents were purchased from eBioscience (San Diego, CA, USA). The staining scheme can be found in the antibody instructions.

### 2.5. Histology

Paraffin-embedded tissue sections were prepared by Servicebio (Wuhan, Hubei, China) and stained with hematoxylin and eosin (H&E). Paraffin-embedded tissue sections (4–5 μm) were subjected to standard hematoxylin and eosin (H&E) staining through sequential steps: Dewaxing in xylene (2 × 5 min), rehydration in graded ethanols (100%, 100%, 95%, 80%, 70%; 2 min each), and distilled water rinse; nuclear staining with Mayer’s hematoxylin (8 min), running-water wash, differentiation in 1% acid alcohol (1–3 s), bluing in 0.1% ammonia water (1 min), and final wash; cytoplasmic counterstaining in 1% eosin Y (90 s); dehydration through graded ethanols (70%, 80%, 95%, 100%, 100%; 1 min each), clearing in xylene (2 × 3 min), and mounting with neutral synthetic resin under coverslips.

The degree of insulitis was scored as follows: no insulitis (absence of cell infiltration), peri-insulitis (infiltration only at the islet periphery), mild insulitis (<50% of the islet area infiltrated), or severe insulitis (≥50% of the islet area infiltrated) [18].

### 2.6. RNA Extraction and Sequencing

Total RNA was extracted from intestinal LPL and splenic lymphocyte samples using the Eastep Super Total RNA Extraction Kit (Promega, Shanghai, China) following the manufacturer’s protocol. Cleared lysates were mixed with 0.5 volumes of absolute ethanol, homogenized by vigorous pipetting (20–25 times), and loaded onto spin columns. After centrifugation (12,000–14,000× *g*, 1 min), columns were washed with 600 μL RNA Wash Buffer (12,000–14,000× *g*, 45 s). On-column DNase I treatment was performed by applying 50 μL of freshly prepared DNase I solution (5 μL 10X DNase I Buffer, 5 μL DNase I, 40 μL nuclease-free water; 15 min incubation at RT). Columns were washed twice with 600 μL RNA Wash Buffer (12,000–14,000× *g*, 45 s each), followed by a final centrifugation (12,000–14,000× *g*, 2 min) to dry membranes. RNA was eluted with 50–200 μL nuclease-free water (2 min incubation at RT; 12,000–14,000× *g*, 1 min) and stored at −70 °C.

RNA quality was assessed using the Agilent 4200 Bioanalyzer (Agilent Technologies, Santa Clara, CA, USA) to determine RNA concentration, RNA integrity number (RIN), 28S/18S ratio, and fragment size. Strand-specific RNA-seq libraries were prepared using the NEBNext^®^ Ultra™ I RNA Library Prep Kit for Illumina (NEB, Ipswich, MA, USA) according to the manufacturer’s guidelines. Library quality was evaluated using the Agilent Bioanalyzer 4200 system, and sequencing was performed on the Illumina Xten platform. RNA sequencing and read alignment against the mouse genome (mm9) were conducted by Nanjing GenMap Technology Co., Ltd. (Nanjing, China). Gene set enrichment analysis was conducted on gene expression data by employing gene set enrichment analysis (GSEA) and hierarchical cluster analysis with Multiexperiment Viewer (MeV 4.9). The RNA sequencing data are available at the Gene Expression Omnibus under SRA accession number PRJNA1127264.

It is worth noting that in the data, SL is the abbreviation of splenic lymphocytes in the treatment group (GAD65 group), and IL is the abbreviation of intestinal LPLs in this group. “C” is the abbreviation of the control group (PBS group). C1 and C2 represent, respectively, the abbreviations of splenic lymphocytes and intestinal LPLs in the control group.

### 2.7. Real-Time RT-PCR

RNA was extracted by means of the Trizol method. Tissues or cells were homogenized in TRIzol™ reagent (1 mL per 50–100 mg tissue/10^7^ cells), incubated at 15–30 °C for 5 min, mixed with 0.2 volumes chloroform, and centrifuged at 12,000× *g* (4 °C, 15 min). The aqueous phase was transferred to a new tube, combined with 0.5 volumes of isopropanol, incubated at –20 °C for 10 min, and centrifuged (12,000× *g*, 4 °C, 10 min) to pellet RNA. Pellets were washed twice with 75% ethanol (centrifugation: 7500× *g*, 4 °C, 5 min), air-dried for 5–10 min, resuspended in nuclease-free water, and treated with DNase I (optional; 15 min, 37 °C) to remove genomic DNA. RNA was stored at –80 °C. Total RNA was reverse-transcribed using the GOScript Reverse Transcription System (Promega, Madison, WI, USA) following the manufacturer’s protocol. Quantitative PCR was performed using the CFX96 Touch Real-Time PCR Detection System (Bio-Rad, Hercules, CA, USA). The qPCR response procedures were as follows: 95 °C for 5 min, followed by 38 cycles of 95 °C for 10 s and 55 °C for 30 s, 72 °C for 30 s. The melting curve was set at 65 °C to 95 °C with an increment of 0.5 °C per cycle for 5 s. Amplification was carried out using GOTaq qPCR Master Mix (Promega, Madison, WI, USA). Melting curve analysis and the 2^−ΔCt^ method were used for data analysis, with GAPDH and U6 as the reference genes. Each group included three independent samples, with each sample analyzed in triplicate. Statistical significance was set at *p* < 0.05. Primer sequences are listed in Supplementary Table S3.

### 2.8. Statistical Analyses

The *t*-test using the Random Variance Model (RVM) was applied to increase the degrees of freedom for small sample sizes when comparing differentially expressed mRNAs between vaccinated and control groups. Differentially expressed genes (DEGs) were identified based on statistical significance and false discovery rate (FDR) assessments, using a predefined threshold. Significant differential expression was defined as *p* < 0.05 and |fold change (FC)| > 2 or |fold change (FC)| > 1 for mRNAs.

## 3. Results

### 3.1. Oral GAD65 L. lactis Alleviated Hyperglycemia and Diabetes

At eight weeks of age, NOD mice were orally immunized with the GAD65 recombinant *L. lactis* vaccine (Figure 1A). Two weeks after the final immunization, basal levels of GAD65-specific IgG antibodies were detected in the serum of NOD mice within the control group. In comparison to the control group, the GAD65 group exhibited a marginal increase in GAD65-specific IgG antibody levels; however, this increase did not reach statistical significance (Figure 1B). Importantly, the GAD65 group demonstrated a significantly elevated level of anti-GAD65 IgA antibodies (Figure 1C). Blood glucose levels were monitored weekly, and two weeks after the final immunization, only mild blood glucose changes (Figure 1D) and insulitis (Figure 1E, Supplementary Figure S1D) were observed in the GAD65 group.

### 3.2. The Difference in Lymphocyte Responses in the Intestinal Lamina Propria and Spleen After Oral GAD65 L. lactis

**Figure 2:**
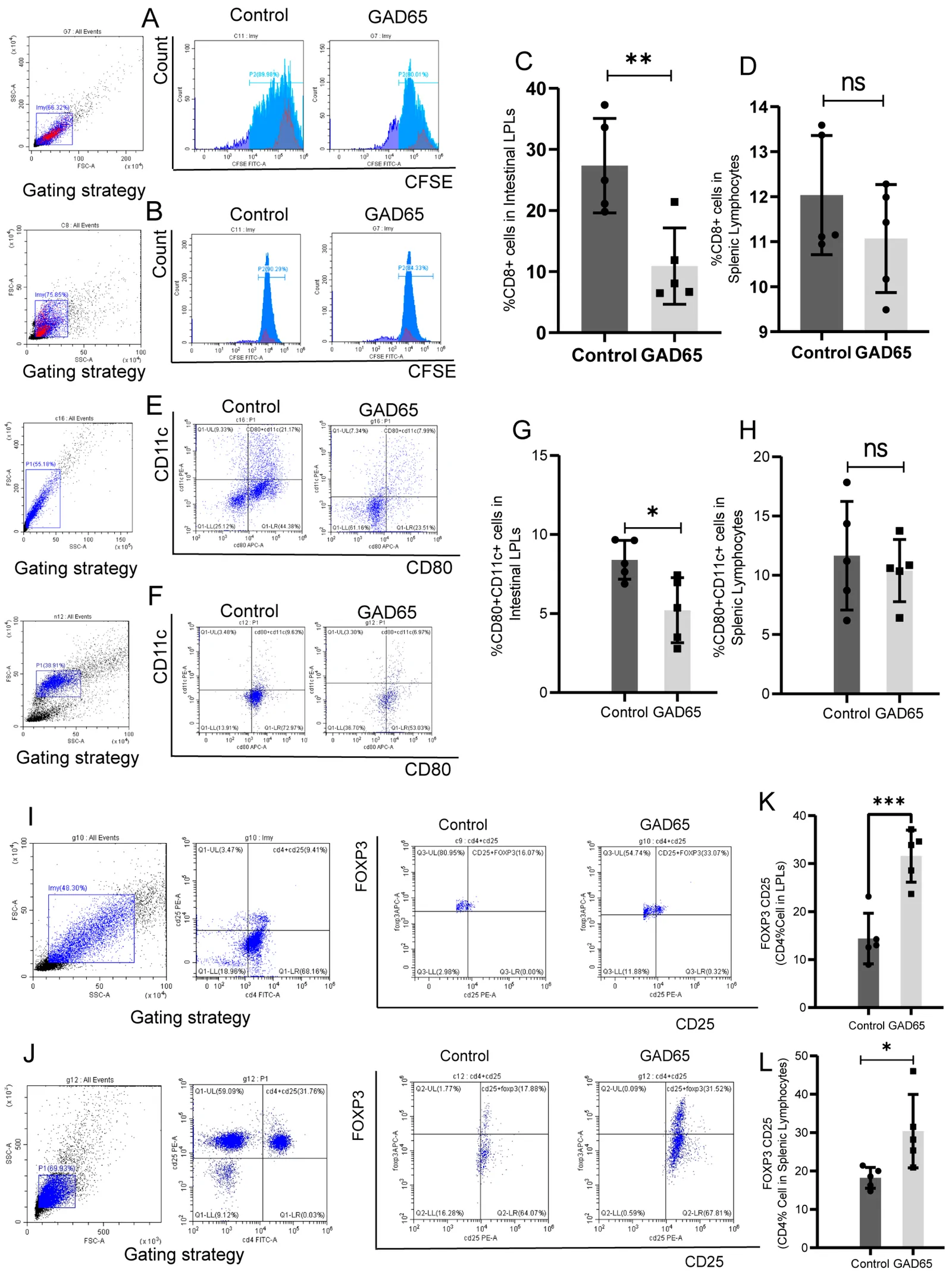
Results of flow cytometry analysis of intestinal LPLs and splenic lymphocytes from NOD mice (*n* = 5). (**A**,**B**) Flow cytometry circle-gate strategy and analysis of GAD65-specific T cell proliferation legend. (**C**,**D**) GAD65-specific T cell proliferation inintestinal LPLs and splenic lymphocytes (** *p* < 0.01, ns *p* > 0.05). (**E**,**F**) Flow cytometry circle-gate strategy and analysis legend for mature DC (CD11c^+^ CD80^+^). (**G**,**H**) Mature DC (CD11c^+^ CD80^+^) content in intestinal LPLs and splenic lymphocytes (* *p* < 0.05, ns *p* > 0.05). (**I**,**J**) Flow cytometry circle-gate strategy and analysis of CD4^+^ CD25^+^ Foxp3^+^ T cell legend. (**K**,**L**) Proportion of CD4^+^ CD25^+^ Foxp3^+^ T cells in CD4^+^ T cell subsets in intestinal LPLs and splenic lymphocytes (*** *p* < 0.0001, * *p* < 0.05).

To evaluate the efficacy of the GAD65 recombinant *L. lactis* vaccine in suppressing the proliferation and differentiation of GAD65-specific CD8^+^ T cells, flow cytometry was performed on splenic lymphocytes and intestinal LPLs two weeks post-immunization. As shown in Figure 2, GAD65-specific T cell proliferation was significantly suppressed in intestinal LPLs from the GAD65 group compared with the control group (Figure 2A,C, *p* < 0.05), whereas no significant differences were observed in splenic lymphocytes (Figure 2B,D). These findings suggest that oral administration of the GAD65 *L. lactis* vaccine effectively inhibited the proliferation of autoreactive T cells in intestinal LPLs.

Furthermore, a significant reduction in mature dendritic cells (DCs) (CD11c^+^ CD80^+^) was observed in intestinal LPLs in the GAD65 group compared with the control group (Figure 2E,G, *p* < 0.05). However, no significant suppression of DC maturation was detected in splenic lymphocytes (Figure 2F,H).

**Figure 3:**
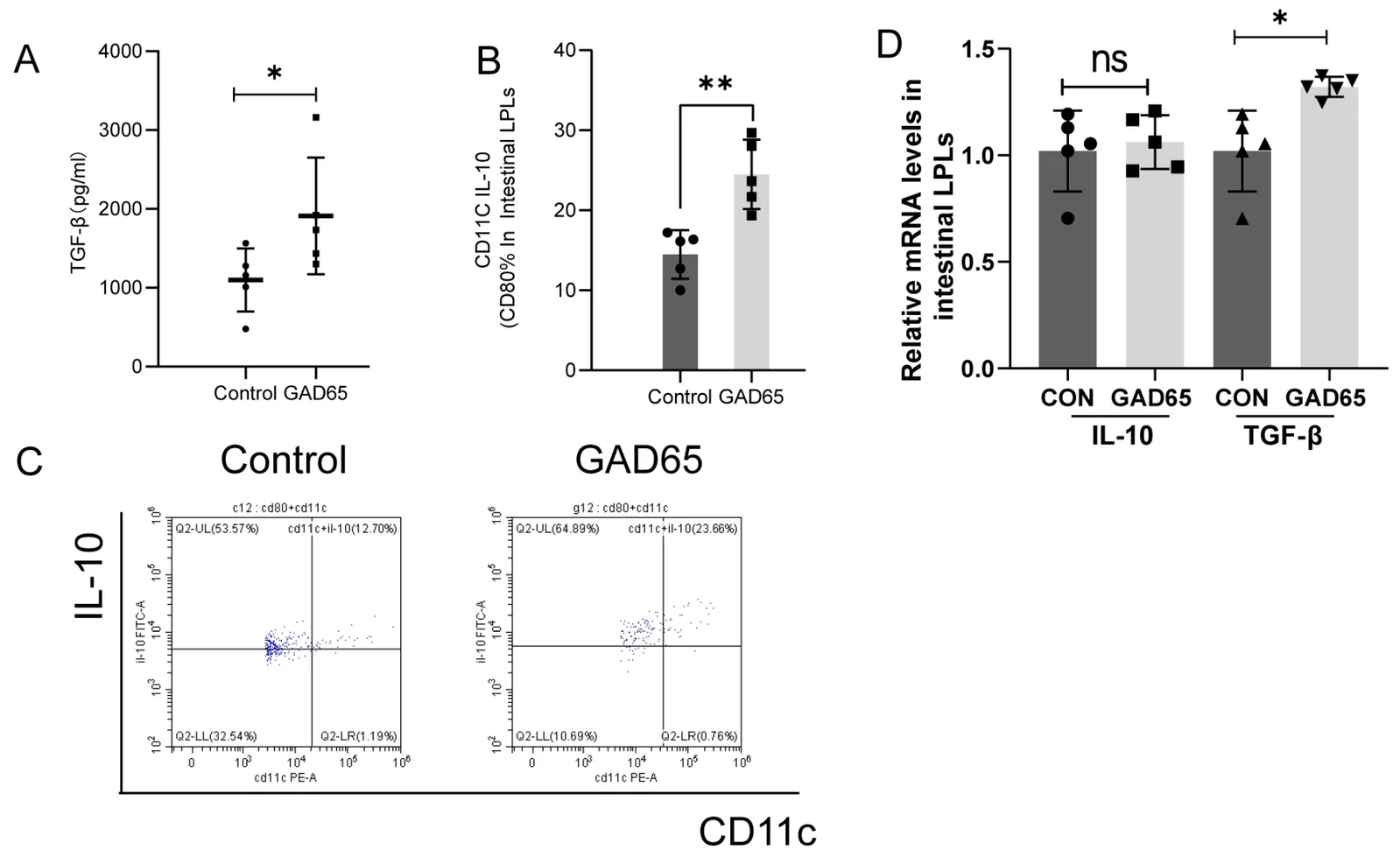
Detection of anti-inflammatory cytokines-related indicators (*n* = 5). (**A**) Detection of TGF-β level in serum (* *p* < 0.05). (**B**) Percentage of immature DC cells (CD80^+^ CD11c^+^) capable of secreting IL-10 in intestinal LPLs (** *p* < 0.005). (**C**) Legend of Flow Cytometry Analysis of DC Cells (CD80^+^ CD11c^+^). (**D**) mRNA content of IL-10 and TGF-β in intestinal LPLs (ns *p* > 0.05, * *p* < 0.05).

Tregs, characterized by CD4, CD25, and the transcription factor Foxp3, play a crucial role in controlling inflammation and reducing autoimmune disease risk [19,20]. In our study, the GAD65 group exhibited a significantly higher proportion of CD4^+^ CD25^+^ Foxp3^+^ T cells within the CD4^+^ T cell subset in both LPL and splenic populations compared with the control group (Figure 2I–L, *p* < 0.05). Moreover, a significant increase in serum TGF-β secretion was observed (Figure 3A, *p* < 0.05). Additionally, IL-10 secretion by immature DCs (CD11c^+^ CD80^+^) in intestinal LPLs significantly increased (*p* < 0.01), as shown in Figure 3B,C. qPCR analysis further confirmed the slight increase of IL-10 mRNA and the significant upregulation of TGF-β mRNA level in intestinal LPLs (Figure 3D, *p* < 0.05).

### 3.3. Oral GAD65 L. lactis Induced Significant Change of Transcriptome in the Intestinal LPLs and Splenic Lymphocytes

To explore the impact of oral immunization on immune responses and differences between intestinal LPLs and splenic lymphocytes, we performed RNA sequencing. Signature transcripts of mRNA and non-coding RNA expressed in intestinal LPLs and splenic lymphocytes were identified (Table 1, Supplementary Figure S2), with more pronounced expression changes in intestinal LPLs; therefore, when conducting the differential screening, we set the criteria as *p*-value FDR < 0.05; |fold change (FC)| > 2 for LPLs; and a fold change (FC) > 1 for splenic lymphocytes was set as the screening criterion.

Hierarchical clustering analysis (Supplementary Figure S2A for mRNAs, Figure S2B for miRNAs, and Figure S2C for lncRNAs) revealed two major clusters. qPCR validation of randomly selected mRNAs, microRNAs, and long non-coding RNAs (Supplementary Figure S3) confirmed consistency with transcriptomic sequencing trends (Supplementary Tables S1–S3).

### 3.4. Oral GAD65 L. lactis Induced Different Biological Processes in the Intestinal LPLs and Splenic Lymphocytes

**Figure 4:**
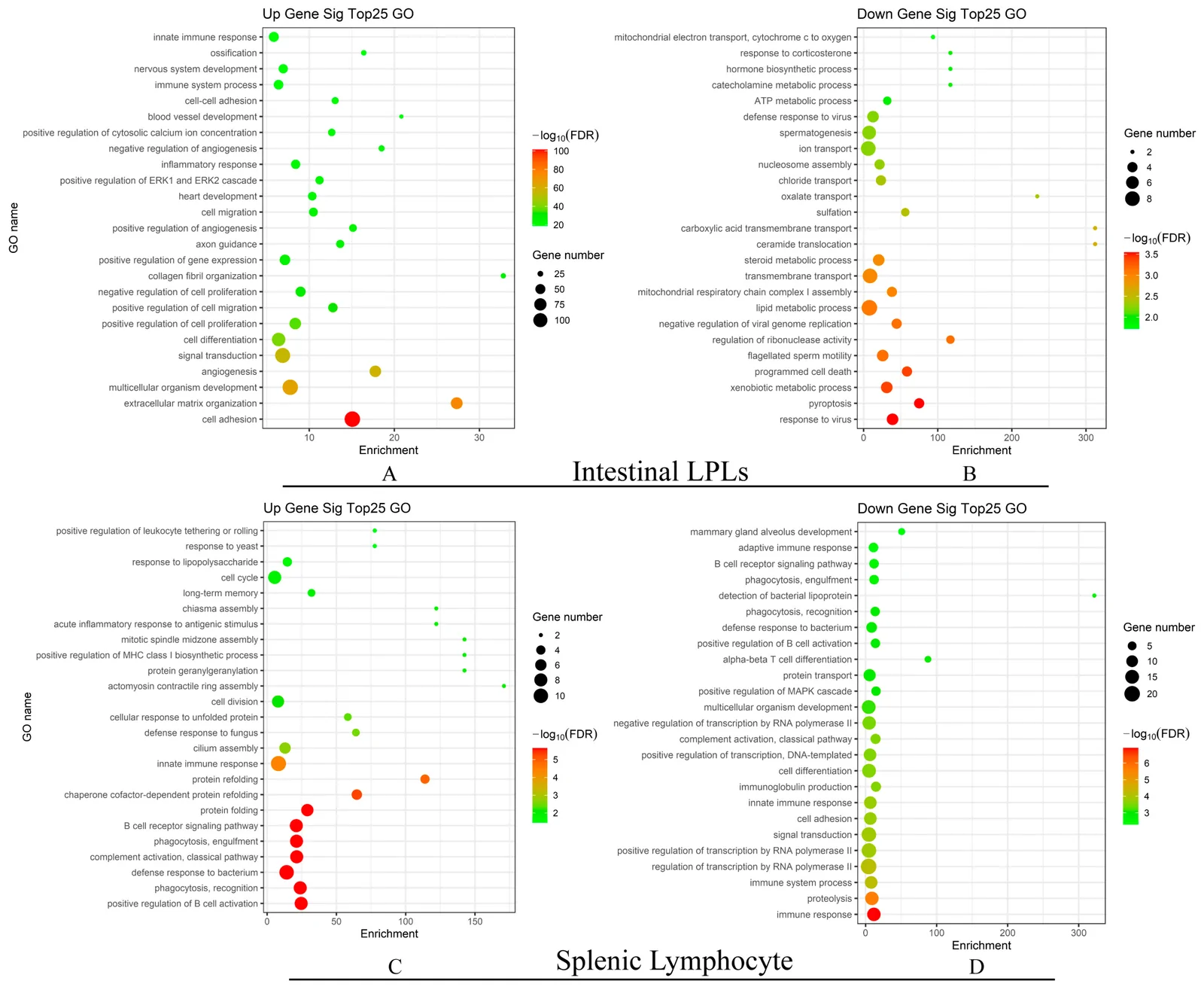
The top 25 significantly enriched GO terms in LPLs and splenic lymphocytes after GAD65 *L. lactis* vaccination (*n* = 3). (**A**,**B**) Up/down gene GO analysis of LPLs. (**C**,**D**) Up/down gene GO analysis of splenic lymphocytes.

**Figure 5:**
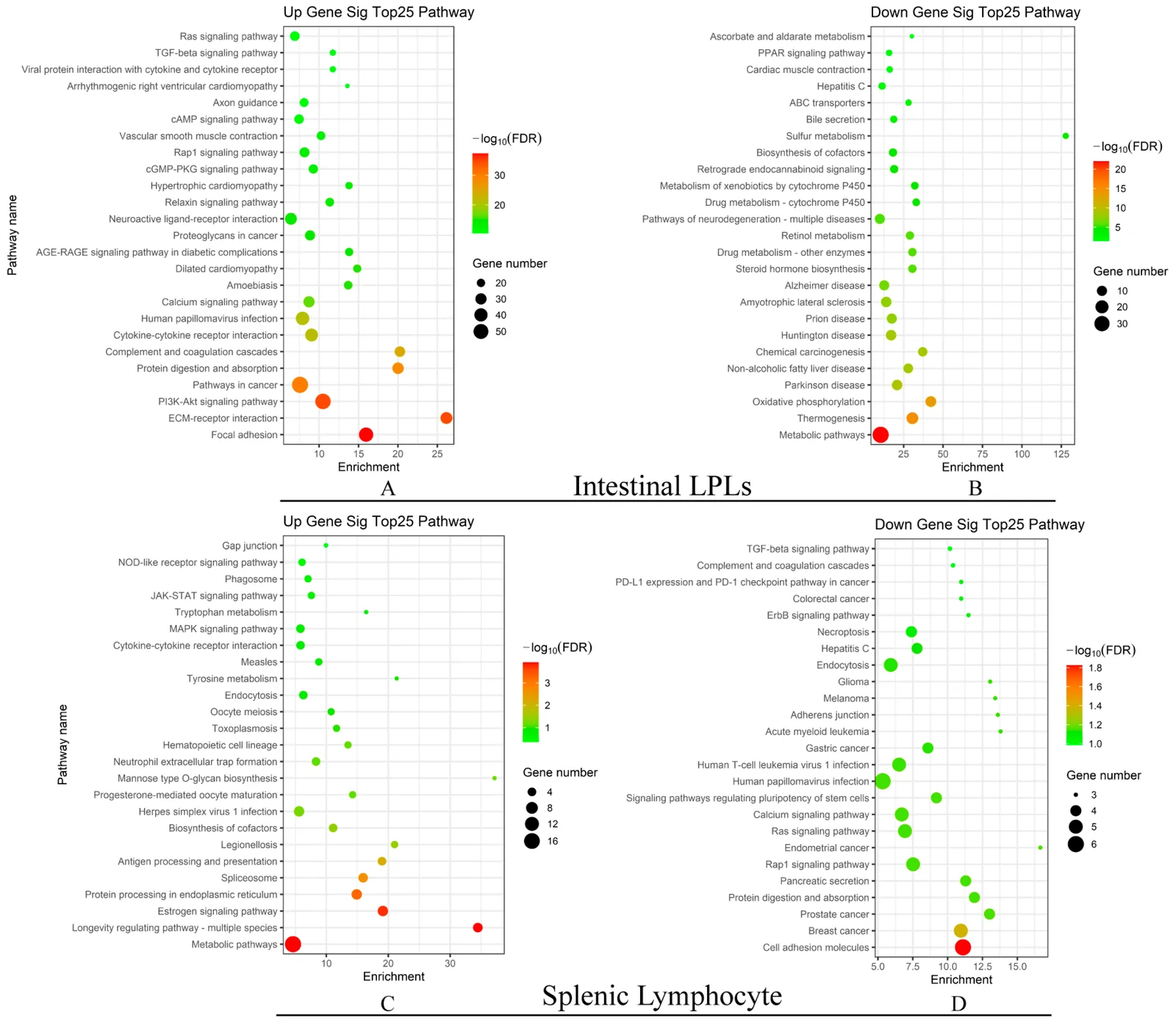
The top 25 significantly enriched KEGG pathway in LPLs and splenic lymphocytes after GAD65 *L. lactis* vaccination (*n* = 3). (**A**,**B**) Up/down gene KEGG analysis of LPLs. (**C**,**D**) Up/down gene KEGG analysis of splenic lymphocytes.

To elucidate the biological processes affected by GAD65 *L. lactis* vaccination, we performed Gene Ontology (GO) and Kyoto Encyclopedia of Genes and Genomes (KEGG) analyses on significantly up- and downregulated genes (*p* < 0.05). The top 25 significant GO and KEGG terms are shown in Figure 4 and Figure 5, respectively.

Upregulated GO terms in intestinal LPLs (Supplementary Table S4) were primarily associated with positive regulation of cell adhesion, while in splenic lymphocytes (Supplementary Table S5), they were related to positive regulation of B cell activation (Table 2). Conversely, downregulated GO terms were linked to viral responses in intestinal LPLs and immune responses in splenic lymphocytes. Notably, upregulated GO terms in intestinal LPLs also included innate immune response, inflammatory response, and immune system processes.

KEGG analysis revealed significant upregulated enrichment in focal adhesion in LPLs (Supplementary Table S6), while metabolic pathways were predominant in splenic lymphocytes (Supplementary Table S7). Conversely, downregulated KEGG terms included cell adhesion molecules in splenic lymphocytes and metabolism pathways in intestinal LPLs (Table 3). The drug metabolism-cytochrome P450, metabolism of xenobiotics by cytochrome P450 were downregulated in intestinal LPLs; however, tyrosine metabolism and tryptophan metabolism were upregulated in splenic lymphocytes. Notably, the TGF-β signaling pathway was upregulated in intestinal LPLs but downregulated in splenic lymphocytes. This finding corroborates our qPCR results regarding TGF-β mRNA levels and the flow cytometry data obtained from intestinal LPLs, suggesting that oral GAD65-*L. lactis* vaccination can induce immune tolerance and promote local immune tolerogenic transcriptomic profiles.

**Figure 6:**
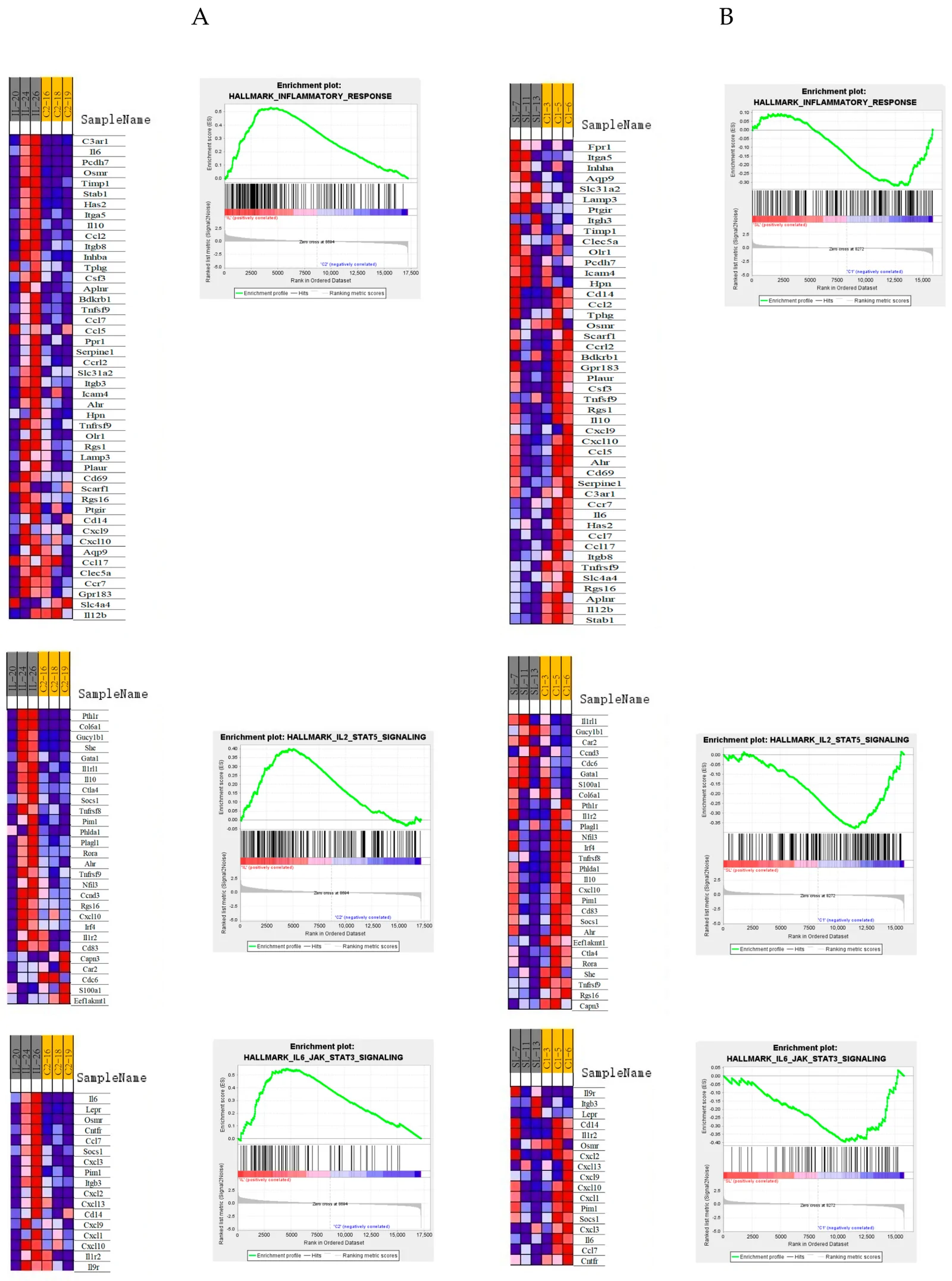
Signaling pathways associated with immune response are differentially regulated between LPLs and splenic lymphocytes using GSEA Hallmake analysis (*n* = 3,|NES| > 1, NOM *p*-val < 0.05, FDR q-val < 0.25). (**A**) LPLs, (**B**) splenic lymphocytes.

Gene Set Enrichment Analysis (GSEA) revealed significant differences in immune responses between intestinal LPLs and splenic lymphocytes. The immune response was significantly enhanced in intestinal LPLs but suppressed in splenic lymphocytes following oral administration of GAD65 *L. lactis* (Supplementary Table S8). Further analysis indicated differential regulation of immune-related signaling pathways. Inflammatory signaling, IL-6-JAK-STAT3, and IL-2-STAT5 pathways were upregulated in intestinal LPLs but downregulated in splenic lymphocytes (Figure 6). Significantly, within the inflammatory response signaling pathway and the IL-2-STAT5 signaling pathway, there was a marked upregulation of inflammatory cytokines, including IL-6, alongside the suppressive cytokine IL-10 in intestinal LPLs. Conversely, these cytokines were notably downregulated in splenic lymphocytes.

### 3.5. Oral GAD65 L. lactis Promoted Different T and B Cell Immune Responses in the Intestinal LPLs and Splenic Lymphocytes

**Figure 7:**
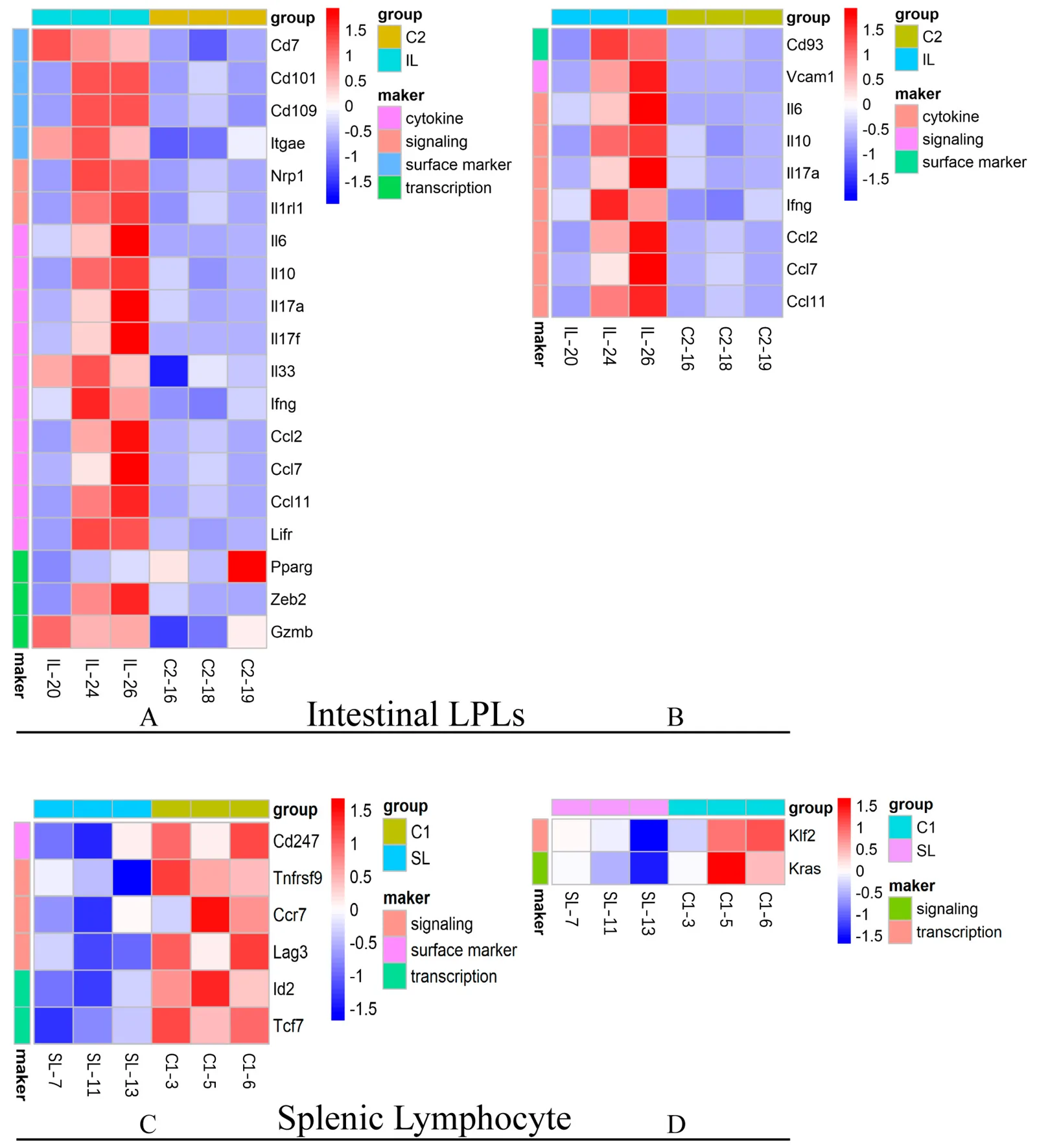
Transcriptional profiles of T- and B-cell-associated cytokines, signal transduction, surface markers, and transcribed genes in LPLs and splenic lymphocytes. (**A**,**B**) LPLs, (**C**,**D**) splenic lymphocytes. (*n* = 3, *p* < 0.05, |Log2 Fold Change| > 1.5).

We further examined the impact of the observed biological processes on adaptive immune responses. Analysis of T and B cell-associated cytokines, signaling pathways, surface markers, and transcription factors revealed distinct transcriptional profiles between intestinal LPLs and splenic lymphocytes (Figure 7). The selected genes have been listed in Supplementary Table S3.

Our findings indicate that oral administration of the *L. lactis* vaccine primarily activated T and B lymphocytes in intestinal LPLs, whereas splenic lymphocytes exhibited minimal activation. This aligns with previous transcriptomic evidence showing greater variability in intestinal LPLs. Notably, the T cell surface marker CD101 was upregulated in intestinal LPLs. Additionally, inflammatory cytokines such as IL-6, IL-17a, and IFN-γ, as well as the suppressive cytokine IL-10, were significantly upregulated in both T and B cells within intestinal LPLs. This observed upregulation of IL-6 and IL-10 aligns with the findings from the GSEA analysis.

Furthermore, chemokines involved in cell recruitment, antigen collection, and inflammatory cell aggregation, including Ccl2, Ccl7, and Ccl11, were upregulated in both T and B cells in intestinal LPLs. In contrast, several genes related to crucial functions of T cells—Cd247, Tnfrs9, Ccr7, Lag3, and Tcf7—exhibited reduced expression in splenic T lymphocytes post-vaccination. Additionally, the expression levels of Kras, a pivotal molecule in signal transduction, and Klf2, which is implicated in the regulation of biological processes including cell differentiation, inflammatory responses, and oxidative stress in B cells, were found to be reduced in splenic lymphocytes.

## 4. Discussion

Oral tolerance therapy is an antigen-specific immunotherapy that has emerged as a promising approach for preventing and treating autoimmune diseases [21,22,23,24]. Extensive research supports the notion that long-term administration of low-dose antigens can effectively suppress Th1-mediated immune responses, which play a crucial role in autoimmune pathogenesis [22,23,24,25]. However, despite their immunological and practical advantages, only a limited number of oral vaccines are currently available [26,27]. This is partly due to the limited understanding of intestinal immunity, particularly oral immunity, despite the intestine being the largest immune organ in the human body.

Since 1991, efforts have been made to induce immune tolerance and antigen-specific Tregs in humans through oral administration of single or combined antigens to lower blood glucose and reverse diabetic characteristics [28]. While some success has been achieved in animal models of T1D [29,30,31,32,33], clinical trials using oral insulin combined with intensive subcutaneous insulin therapy have not been effective. One major limitation is that oral antigens undergo degradation in the intestinal lumen and are poorly presented to antigen-presenting cells (APCs), thereby failing to induce mucosal immune tolerance. To overcome this challenge, various delivery vectors based on plants, microbes, or nanoparticles (NPs) have been developed to protect autoantigens from degradation and enhance antigen presentation within gut-associated lymphoid tissue (GALT).

*L. lactis* is a widely recognized safe microorganism found in humans, animals, plants, and the environment. Studies have demonstrated that *L. lactis* can be engineered to express various foreign protein molecules, including antigens, enzymes, cytokines, antibodies, and allergens [13,14,15]. These proteins are continuously released as *L. lactis* propagates in the mucosal tissues of the gastrointestinal, respiratory, and urinary tracts. It is notable that even the expression of cytoplasmic antigens in lactic acid bacteria can induce an immune response [34,35]. The lamina propria plays a key role in promoting systemic and mucosal immune tolerance upon antigen absorption, a property exploited in experimental oral immunotherapies for autoimmune diseases and allergies [21].

Building on these properties, we utilized *L. lactis* to express the GAD65 autoantigen, which not only protected the vaccine from degradation in the intestinal environment but also enhanced its recognition by intestinal immune cells. Furthermore, we verified that the recombinant lactic acid bacteria could colonize and survive in the intestine after oral administration to confirm the survival ability of the bacteria after ingestion.

In our study, we identified the presence of GAD65-specific IgG antibodies in the serum of NOD mice within the control group, indicating that these mice exhibit basal levels of GAD65-specific IgG antibodies. Consequently, when we assessed the levels of GAD65-specific IgG antibodies two weeks after the final immunization, there was no significant increase observed in the GAD65 group. Nonetheless, a notable elevation in the level of GAD65-specific IgA antibodies was detected in the GAD65 group, suggesting that oral administration of the GAD65 recombinant *L. lactis* vaccine preferentially induces GAD65-specific secretory IgA (sIgA) production in the intestinal mucosa. It is indicated that, within the NOD mouse model, the GAD65 antibody serves not only as a critical biomarker for autoimmune diabetes but also as a significant target for immune intervention. One valuable attribute of *L. lactis* as bacterial delivery vehicles for vaccines lies in their potential to trigger antigen-specific secretory IgA responses at mucosal surfaces [10]. sIgA regulates the mucosal immune response by modulating DC activation and contributing to immune tolerance [36]. This stimulation not only helps maintain intestinal homeostasis but also plays a critical role in preventing inflammation and autoimmune responses.

Two weeks after completing oral immunization, we observed a remission in disease progression. The vaccine suppressed early inflammation and maintained stable blood glucose levels in NOD mice, consistent with previous studies involving oral administration of GAD65 or insulin in NOD mice [37,38]. This effect was primarily mediated by the induction of intestinal GAD65-specific CD11c^+^ IL-10^+^ DCs, which subsequently promoted CD4^+^ CD25^+^ Foxp3^+^ Treg differentiation and TGF-β secretion, thereby inhibiting CD8^+^ effector T cell proliferation.

We observed distinct differences in immune responses between distal splenic lymphocytes and intestinal lymphocytes during oral immunization, which appeared somewhat inconsistent. Notably, only CD4^+^ CD25^+^ Foxp3^+^ Treg cells significantly increased in both splenic lymphocytes and intestinal LPLs. Oral immune tolerance is a robust and long-lasting process with both local and systemic effects, where iTregs and DCs play a crucial role in mucosally induced suppression. The site of antigen entry significantly influences the immune response. Recent studies [39] suggest that oral antigen administration leads to the formation of specific germinal centers not only in Peyer’s patches (PP) and mesenteric lymph nodes (mLN) but also in the spleen [40]. This indicates that different lymphoid compartments can be influenced by antigen entry location, resulting in variable immune reactivity. Given the heterogeneous cellular composition of the LPL compartment—which includes regulatory T cells (Tregs), dendritic cells (DCs), and innate lymphoid cells (ILCs)—further investigation is needed to clarify how their gene expression profiles and functional states differ from those in the spleen.

The spleen, a critical secondary lymphoid organ, plays a pivotal role in filtering and monitoring the bloodstream for foreign substances such as bacteria, viruses, and antigens [41]. As a direct connection to the circulatory system, it serves as a central hub where immune cells encounter potential threats [42]. Some studies suggest that orally ingested antigens reach the spleen via specialized M cells in intestinal tissue [43]. These M cells transport antigens from the intestinal lumen into underlying lymphoid tissues, where they interact with immune cells. Another hypothesis posits that DCs in both GALT and splenic red pulp capture orally administered antigens and migrate to the spleen, where they present antigenic fragments to T cells, initiating an adaptive immune response. However, the precise role of the spleen in responding to orally administered antigens remains unclear and warrants further investigation. Understanding spleen involvement in mucosal immunity has important implications for vaccine development strategies targeting mucosal surfaces.

Comprehensive immune assessment and GO analysis demonstrated that oral immunization induced immune responses in both the intestine and splenic lymph nodes. Among these responses, the most significantly upregulated GO terms were associated with the positive regulation of B cell activation in the spleen and cell adhesion in the intestine. In contrast, the most significantly downregulated GO terms were related to immune response in the spleen and viral response in the intestine. In the intestine, *L. lactis* plays a crucial role in enhancing the cell adhesion signaling pathway, as well as mucosal inflammation and immune response. Studies suggest that *L. lactis* modulates inflammatory cytokine production and promotes anti-inflammatory responses in the intestine [44]. In the study by Takiishi et al. [45], it was pointed out that diabetes reversal induced by *L. lactis*-based therapy is accompanied by and dependent on the generation of functional Foxp3^+^ Tregs. Interestingly, despite triggering B cell activation in the spleen, overall immune responses appeared to be suppressed. This finding suggests that while the *L. lactis* recombinant vaccine promotes specific immune functions, it may also exert regulatory effects to prevent excessive or unnecessary immune activation. Overall, GO analysis highlights how oral recombinant *L. lactis* vaccine influences both local (intestinal) and systemic (splenic) immune responses. By enhancing cell adhesion signaling in the intestine and regulating inflammatory processes, the recombinant *L. lactis* vaccine contributes to intestinal health while supporting appropriate immune responses throughout the body.

KEGG analysis revealed that the TGF-β signaling pathway was upregulated in the intestine but downregulated in the spleen, whereas metabolic pathways were upregulated in the spleen but downregulated in the intestine. Numerous studies have demonstrated a strong correlation between intestinal metabolism and immune response. The amino acids tyrosine (Tyr) and tryptophan (Trp) play pivotal roles in regulating energy metabolism and suppressing proinflammatory cytokine production in splenic cell lysates [46]. Notably, Trp significantly suppressed the production of IL-1β, IL-6, IL-10, IL-17A, and GM-CSF while inducing IFN-γ synthesis, an effect most pronounced in spleen cell lysates [47]. Conversely, tryptophan metabolism within the intestinal microbiome generates aryl hydrocarbon receptor (AhR) agonists, which strongly activate AhR, promoting the induction of tolerogenic DCs and Tregs [47,48,49,50]. This suggests a potential mechanism through which tryptophan metabolites in the intestine modulate immune responses and contribute to immune homeostasis. These findings underscore the differential regulation of pathways across organs such as the intestine and spleen. Abnormalities in the gut-spleen axis may be involved in diseases, such as abnormal activation of splenic lymphocytes in patients with inflammatory bowel disease (IBD) [51,52]. Further investigation into these interactions may provide insights for targeted interventions in metabolic disorders and immune dysfunctions associated with pathway imbalances.

The immunological effects observed in intestinal LPLs and splenic lymphocytes suggest that the vaccine exerts a dual effect: enhancing intestinal LPL activity, which is crucial for intestinal homeostasis and immune regulation, while concurrently suppressing splenic lymphocyte responses. This finding is consistent with previous results, highlighting the complex interplay between vaccines and different immune compartments. These observations suggest that oral administration of the *L. lactis* recombinant vaccine induces an immunosuppressive state in intestinal immune cells in NOD mice. This observation is consistent with the findings of earlier studies conducted by Luerce TD et al. [53], which utilized *Lactococcus lactis* NCDO 2118 in the treatment of dextran sulfate (DSS)-induced colitis models. In these studies, IL-6 production in the intestine was found to potentially enhance mucosal repair and stimulate IL-10 production in the colon. Additionally, there was a significant increase in the number of CD4^+^ CD25^+^ LAP^+^ regulatory T cells in the mesenteric draining lymph nodes and spleen. This is consistent with our GSEA results, which demonstrate the upregulation of IL-6 and IL-10 in intestinal LPLs. Understanding these complex mechanisms is critical for developing targeted therapies to modulate immune responses in autoimmune diseases and provides valuable insights for designing effective oral vaccines.

Heatmap analysis of RNA-seq data provided additional insights into the role of T and B cells in immune regulation. Peripheral T and B cells were primarily engaged in this process, highlighting their essential role in maintaining immune balance. Among the molecules involved in oral tolerance, Ccl7 was particularly noteworthy as a chemokine responsible for preserving immunotolerance by regulating immune cell recruitment and activation in peripheral tissues [54]. Another key molecule, IL-10, is a versatile cytokine with broad anti-inflammatory effects. IL-10 plays a crucial role in sustaining peripheral tolerance by promoting the generation and regulation of adaptive Tregs. The upregulation of these molecules suggests that initial antigen-driven T cell activation in intestinal LPLs following oral vaccination is subsequently modulated through T cell surface receptors and self-secreted cytokines, facilitating either proliferation or tolerance formation.

Through transcriptomic analysis, we suggest that intestinal LPLs adopt a distinct tolerant state following oral vaccination. This state appears to inhibit the maturation of antigen-specific CD80^+^ CD11c^+^ dendritic cells, promote the generation of CD4^+^ CD25^+^ Foxp3^+^ regulatory T cells, and suppress the proliferation of CD8^+^ T cells. The induction of this tolerant state is mediated by TGF-β, IL-10, and various signaling pathways, including metabolic signals. Simultaneously, the spleen plays a crucial role in maintaining a balance between B cell responses and systemic immune regulation.

This study aims to analyze these synergistic mechanisms to enhance our understanding of mucosal and systemic coordination. However, additional functional research into these mechanisms is necessary to improve the design of oral vaccines, addressing current deficiencies and guiding future research directions. For instance, how can the efficacy of cells migrating to the spleen be enhanced following activation by intestinal LPLs to strengthen systemic immune memory? The development of transmucosal and systemic delivery systems is crucial to optimize both local and systemic protection afforded by oral vaccines. Current studies on Mn@mRNA-LNP vaccines [55], wherein manganese ions (Mn^2+^) activate the STING pathway to enhance DC maturation and antigen presentation efficiency, suggest potential applications in oral vaccines to boost mucosal and systemic immune responses. Yale Yue et al. [56] used genetically modified symbiotic bacteria to produce in situ tumor antigen-fused outer membrane vesicles (OMVs) to deliver stimulatory molecules, which could be used to develop other oral vaccines and therapies. Furthermore, addressing safety concerns in vaccine design is imperative, as oral vaccines may induce intestinal inflammation or excessive systemic immune reactions. This requires a balance between adjuvant activation and tolerogenic signals.

## 5. Conclusions

In conclusion, this study demonstrated that oral administration of the GAD65-*L. lactis* vaccine effectively induced immune tolerance and inhibited the progression of T1D. The vaccine’s impact was evidenced by a transcriptomic profile predominantly characterized by mucosal immune tolerance, with secondary effects on systemic immune regulation. These findings offer novel insights into the mechanisms underlying the efficacy of oral vaccines for T1D. The interaction between splenic lymphocytes and intestinal LPLs provides a foundational “bridge” for the design of oral vaccines.

## Figures and Tables

**Table 1:** The number of differentially expressed transcripts between vaccinated and control.

Cell	Comparison	Upregulated	Downregulated	Total
Splenic lymphocytes	lncRNA Vaccinated vs. Control	467	371	838
	miRNA Vaccinated vs. Control	7	5	12
	mRNA Vaccinated vs. Control	176	233	409
intestinal LPLs	lncRNA Vaccinated vs. Control	600	357	957
	miRNA Vaccinated vs. Control	46	17	63
	mRNA Vaccinated vs. Control	1055	162	1217

*n* = 3, *p*-value FDR < 0.05; |fold change (FC)| > 2 for LPLs; |fold change (FC)| > 1 for splenic lymphocytes.

**Table 2:** The list of GO with member genes in vaccinated vs. control group (the top 5 GO pathway).

Upregulated							
Tissue	GO	GO Pathway	No. of Difference Gene	Tissue	GO	GO Pathway	No. of Difference Gene
spleen	GO:0050871	positive regulation of B cell activation	8	intestinal tract	GO:0007155	cell adhesion	124
	GO:0006910	phagocytosis, recognition	8		GO:0030198	extracellular matrix organization	69
	GO:0042742	defense response to bacterium	10		GO:0007275	multicellular organism development	122
	GO:0006958	complement activation, classical pathway	8		GO:0001525	angiogenesis	66
	GO:0006911	phagocytosis, engulfment	8		GO:0007165	signal transduction	115
Downregulated							
spleen	GO:0006955	immune response	14	intestinal tract	GO:0009615	response to virus	5
	GO:0006508	proteolysis	14		GO:0070269	pyroptosis	4
	GO:0002376	immune system process	12		GO:0006805	xenobiotic metabolic process	5
	GO:0006357	regulation of transcription by RNA polymerase II	20		GO:0012501	programmed cell death	4
	GO:0045944	positive regulation of transcription by RNA polymerase II	17		GO:0030317	flagellated sperm motility	5

**Table 3:** The list of KEGG pathways with member genes in vaccinated vs. control group (the top 5 KEGG pathways).

Upregulated							
Tissue	KEGG	KEGG Pathway	No. of Difference Gene	Tissue	KEGG	KEGG Pathway	No. of Difference Gene
spleen	ko1100	Metabolic pathways	17	intestinal tract	ko04510	Focal adhesion	46
	ko 04213	Longevity regulating pathway—multiple species	5		ko04512	ECM-receptor interaction	33
	ko 04915	Estrogen signaling pathway	6		ko04151	PI3K-Akt signaling pathway	54
	ko 04141	Protein processing in the endoplasmic reticulum	6		ko05200	Pathways in cancer	59
	ko03040	Spliceosome	5		ko04974	Protein digestion and absorption	31
Downregulated							
spleen	ko04514	Cell adhesion molecules	6	intestinal tract	ko01100	Metabolic pathways	35
	ko05224	Breast cancer	5		ko04714	Thermogenesis	15
	ko5215	Prostate cancer	4		ko00190	Oxidative phosphorylation	12
	ko04974	Protein digestion and absorption	4		ko05012	Parkinson disease	11
	ko04972	Pancreatic secretion	4		ko04932	Non-alcoholic fatty liver disease	9

## Data Availability

The original contributions presented in this study are included in the article/supplementary material. Further inquiries can be directed to the corresponding authors.

## Supplementary Materials

The following supporting information can be downloaded at: https://www.mdpi.com/article/10.3390/vaccines13090927/s1. Supplementary Figure S1. Recombinant plasmid profile and expression validation of GAD65 *L. lactis* vaccine. (**A**) Plasmid profile of pNZ8148 vector containing codon-optimized GAD65 gene. (**B**) *L. lactis* containing the recombinant pNZ8148 plasmid was able to cytoplasmically (“C”) express and secrete (“S”) the GAD65 protein in response to nisin stimulation at different concentrations. The recombinant GAD65 *L. lactis* was lysed by lysozyme at 52 °C for 2 h. Samples were boiled and loaded onto the gel. Then, the lysate samples were diluted and separated by 10% SDS-PAGE. Rabbit anti-GAD65 (Proteintech, Chicago, IL, USA) was used for immunoreactions. (**C**) The colonization and survival of the GAD65 *L. lactis* vaccine in the intestine (*p* < 0.005). (**D**) Histopathological sections of NOD mice two weeks after the last oral immunization, in which the degree of insulitis progressively increased from left to right in each group, corresponding to the statistical results in Figure 1E. Supplementary Figure S2. Signature transcripts of mRNA/non-coding RNAs expressed by LPLs and splenic lymphocytes. Supplementary Figure S3. Results of validation of expression levels of randomly selected mRNAs/miRNAs/LncRNAs using qPCR. Supplementary Table S1. Differentially transcriptional profiling result of LPLs. Supplementary Table S2. Differentially transcriptional profiling result of splenic lymphocytes. Supplementary Table S3. Primer sequences for qPCR and T- and B-cell-related genes. Supplementary Table S4. Upward/downward GO terms in LPLs. Supplementary Table S5. Upward/downward GO terms in splenic lymphocytes. Supplementary Table S6. Up-/downregulated gene pathways and gene enrichment in LPLs. Supplementary Table S7. Up-/downregulated gene pathways and gene enrichment in splenic lymphocytes. Supplementary Table S8. Analysis Report of Hallmark Gene Sets of LPLs and Splenic Lymphocytes.

## Abbreviations

The following abbreviations are used in this manuscript:

GAD65	Glutamic Acid Decarboxylase 65
LPLs	Intestinal Lamina Propria Lymphocytes
NOD	Non-Obese Diabetic
T1D	Type 1 Diabetes
DCs	Dendritic Cells
Tregs	Regulatory T Cells
ELISA	Enzyme-Linked Immunosorbent Assay
PBS	Phosphate-Buffered Saline
FBS	Fetal Bovine Serum
RIN	RNA Integrity Number
GO	Gene Ontology
KEGG	Kyoto Encyclopedia of Genes and Genomes
GSEA	Gene Set Enrichment Analysis
CFSE	Carboxyfluorescein Succinimidyl Ester
OD	Optical Density
HRP	Horseradish Peroxidase
TGF-β	Transforming Growth Factor-β
sIgA	Secretory Immunoglobulin A
GALT	Gut-Associated Lymphoid Tissue
AhR	Aryl Hydrocarbon Receptor
PP	Peyer’s Patches
mLN	Mesenteric Lymph Nodes
SL	Splenic Lymphocytes in GAD65 group
IL	Intestinal LPLs in GAD65 group
RVM	Random Variance Model
DSS	Dextran Sulfate Sodium
