# Supplementary figures and images for: Transcriptomic Analysis of Immune Tolerance Induction in NOD Mice Following Oral Vaccination with GAD65-Lactococcus lactis
Source: Vaccines (Basel). 2025 Aug 30;13(9):927. doi: 10.3390/vaccines13090927 (PMC12474109; doi:10.3390/vaccines13090927)

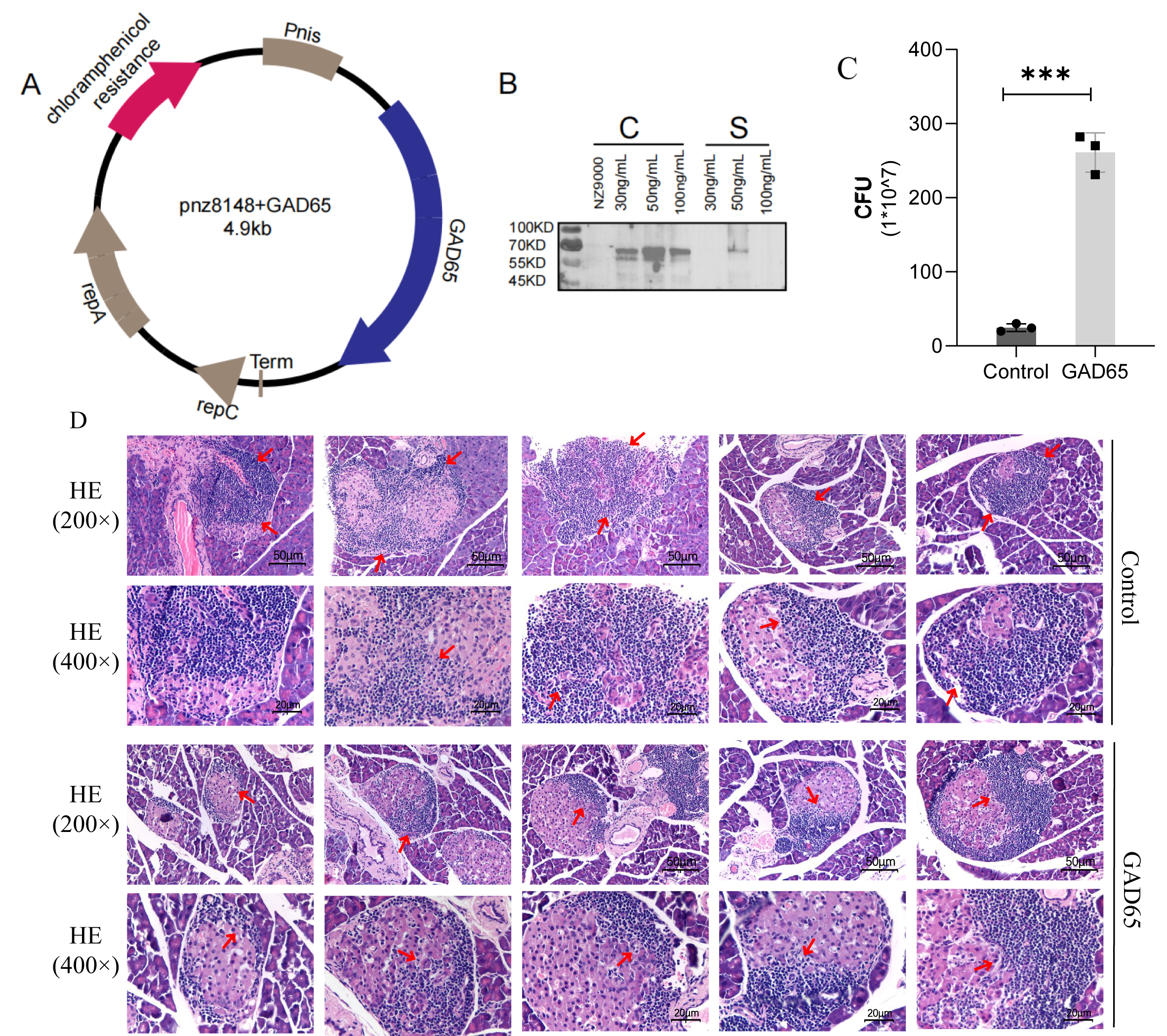

Supplement: Supplementary file 1 [file vaccines-13-00927-s001.zip › Figure S1.tif]

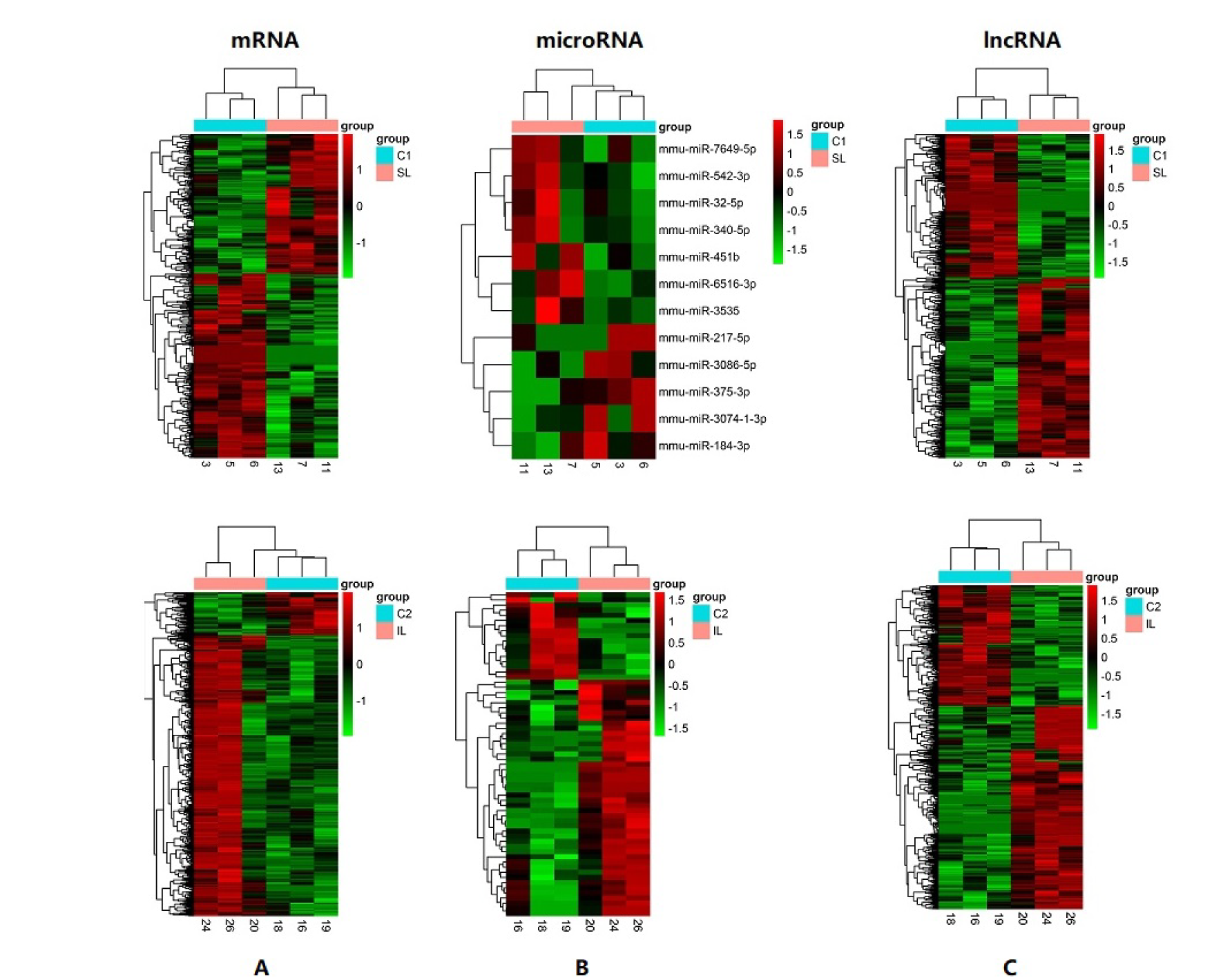

Supplement: Supplementary file 1 [file vaccines-13-00927-s001.zip › Figure S2.TIF]

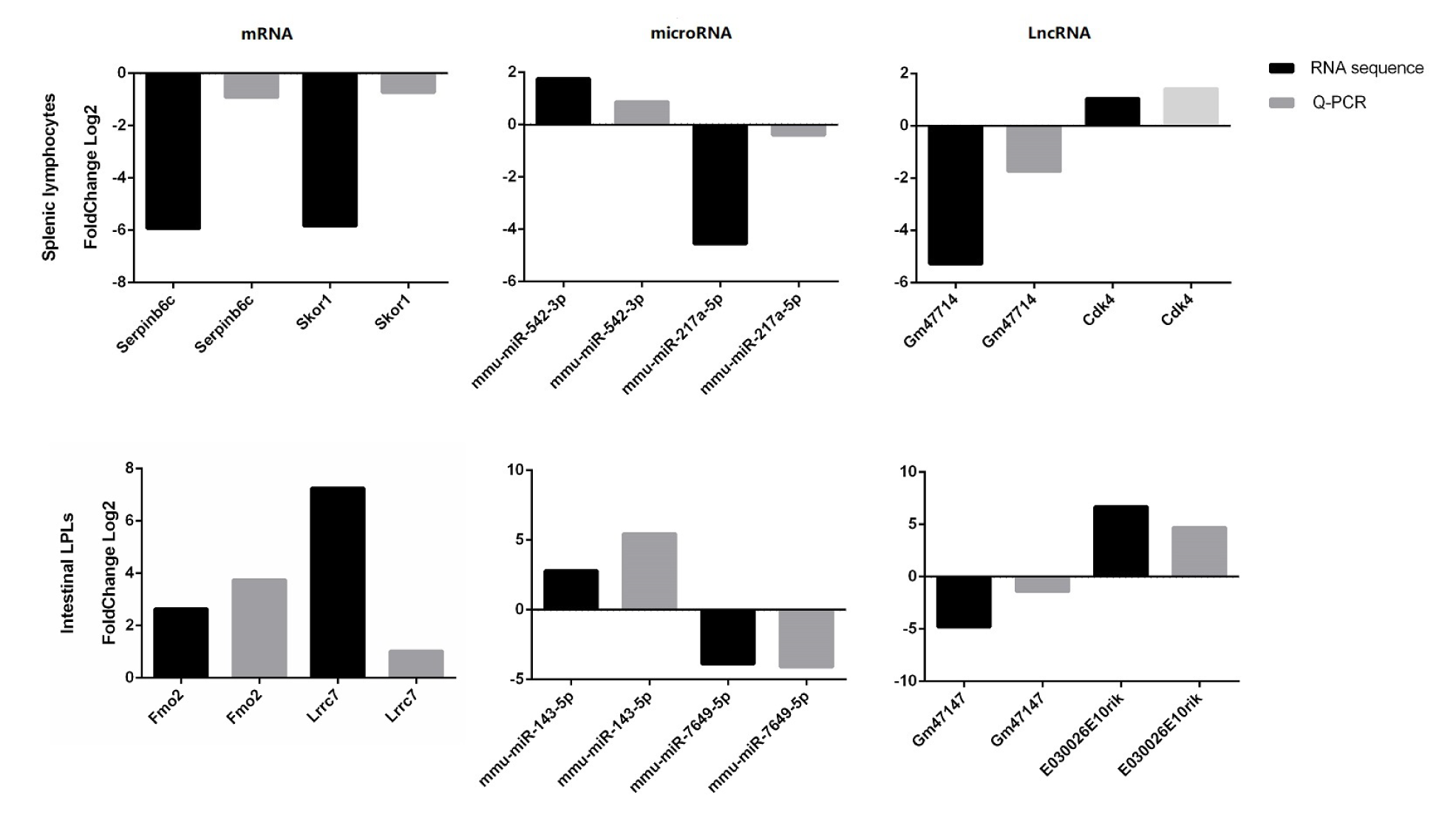

Supplement: Supplementary file 1 [file vaccines-13-00927-s001.zip › Figure S3.TIF]
